# Absorption and Safety With Sustained Use of RELiZORB Evaluation (ASSURE) Study in Patients With Cystic Fibrosis Receiving Enteral Feeding

**DOI:** 10.1097/MPG.0000000000002110

**Published:** 2018-08-01

**Authors:** John Stevens, Colby Wyatt, Perry Brown, Dhiren Patel, Danica Grujic, Steven D. Freedman

**Affiliations:** ∗ Riley Hospital for Children, Indianapolis, IN; † Maine Medical Center, Portland, ME; ‡ St. Luke's Health System, Boise, ID.; § Division of Gastroenterology and Department of Pediatrics Saint Louis University School of Medicine, St. Louis, MO; || Alcresta Therapeutics, Inc, Warren, NJ; ¶ Department of Medicine and Division of Translational Research, Beth Israel Deaconess Medical Center, Harvard Medical School, Boston, MA.

**Keywords:** exocrine pancreatic insufficiency, fat malabsorption, long-chain polyunsaturated fatty acids, omega-3 index, pancreatic enzyme replacement therapy

## Abstract

**Objectives:**

Pancreatic insufficiency (PI) and malabsorption of fats lead to reduced caloric intake, inability to maintain weight, and increased gastrointestinal symptoms. Thus, enteral nutrition (EN) is used in patients with cystic fibrosis (CF) and poor nutritional status. The current study evaluated safety, tolerability, and improvement of fatty acid (FA) status in red blood cell (RBC) membranes, a marker of long-term FA absorption, with an in-line digestive cartridge (RELiZORB) that hydrolyzes fat in enteral formula.

**Methods:**

Patients with CF receiving EN participated in a multicenter, 90-day open-label study during which RELiZORB was used with overnight EN. The primary endpoint was change over time in RBC uptake of docosahexaenoic acid (DHA)+ eicosapentaenoic acid (EPA). Gastrointestinal symptoms were collected to evaluate safety and tolerability. Several clinical and anthropometric parameters were also assessed throughout the study.

**Results:**

A total of 36 subjects completed the study with a mean age of 13.8 years, body mass index of 17.7 and 6.2 years mean use of overnight EN. Fat absorption significantly improved as shown by increased RBC levels of DHA+EPA, improved ω-6/ω-3 ratio, and increased plasma levels of DHA+EPA. RELiZORB use was not associated with any unanticipated adverse events.

**Conclusions:**

RELiZORB use was found to be safe, well tolerated, and resulted in increased levels of FAs in RBCs and plasma. This is the first prospective study to show EN can improve FA abnormalities in CF. Because improvement in omega-3 levels has been shown to help pulmonary and inflammatory status as well as anthropometric parameters in CF, RELiZORB may have important long-term therapeutic benefits in patients with CF.

**What Is Known**

- Approximately 10% to 12% of patients with cystic fibrosis are not meeting their nutritional goals with dietary intake alone requiring them to turn to overnight enteral tube feeding.
- There are no long-term prospective trials in patients with cystic fibrosis to inform treatment regarding enteral nutrition.
- No recommendations can be made regarding the use of pancreatic enzyme replacement therapy with enteral nutrition due to the absence of well-controlled randomized clinical trials.

**What Is New**

- Long-term use of an in-line digestive enzyme cartridge (RELiZORB) with a regular overnight enteral nutrition regimen normalized plasma omega-3 levels and resulted in a 2-fold increase in omega-3 index, a long-term measure of fat absorption in patients with CF.
- RELiZORB use for 90-day period was associated with a lower ratio of omega-6/omega-3 fatty acids, a key marker of inflammation.

Exocrine pancreatic insufficiency (EPI) occurs when pancreatic enzyme activity in the intestinal lumen is low enough that normal digestion cannot occur (1,2). EPI is an important clinical sequela of various developmental and acquired pancreatic conditions, including preterm birth, cystic fibrosis (CF), pancreatitis, pancreatic cancer, pancreatic surgery, and aging (2,3). Pancreatic lipase insufficiency results in maldigestion of dietary fat, causing most of the clinically important EPI symptoms, and complications, including malnutrition (1,3). Up to 90% of patients with CF exhibit some degree of EPI requiring the use of replacement digestive enzyme therapy to assist with the digestion of oral meals and snacks (4).

Beyond clinical gastrointestinal (GI) symptoms, fat malabsorption in EPI also causes deficiencies of long-chain polyunsaturated fatty acids (LCPUFAs), including docosahexaenoic acid (DHA) and eicosapentaenoic acid (EPA) (5–8). LCPUFAs have well-established clinical benefits, including visual, cognitive, anti-inflammatory, and cardiovascular effects, and there is increasing evidence that LCPUFAs are important to the health of patients with CF. DHA and EPA counteract the inflammatory responses of CF and are essential to brain development and cognition (7,9–12). Low levels of DHA may contribute to increased inflammation in patients with CF (6,7,9,10). Evidence from small studies of patients with CF given DHA and EPA supplements indicate improved pulmonary function and decreased pulmonary exacerbations with increased DHA and EPA erythrocyte levels (10,13). Given the increasing recognition of their importance in normal tissue development and health as well as disease severity, LCPUFAs are being targeted as therapeutic interventions to improve patient outcomes (5–7,10).

In patients with EPI, meals and snacks are typically supplemented with oral pancreatic enzyme replacement therapy (PERT) to improve fat absorption (4,14). Unfortunately, oral PERT is not formulated nor indicated for use with continuous enteral tube feedings (15). Because PERT is taken orally during waking hours and is not delivered continuously to the small intestine during tube feeding, it is not ideal for use with continuous enteral nutrition (EN), especially when given overnight. Furthermore, given an absence of evidence from clinical trials, guidelines for EN use in patients with CF make no specific recommendations for the use of PERT with enteral feedings (16). Although PERT remains critical for oral feedings, finding a way to improve fat absorption during enteral tube feedings is critical to improving the nutritional status of patients with CF and EPI.

RELiZORB (immobilized lipase) cartridge (Alcresta Therapeutics, Inc, Newton, MA) is a new therapeutic approach to improve fat absorption in patients who receive EN tube feedings (5,15). It is FDA-approved for pediatric (≥5 years of age) and adult patients to hydrolyze fats found in enteral formula. RELiZORB is a single-use digestive enzyme cartridge and connects in-line with EN feeding sets (17). Lipase is covalently bound to small polymer beads to form a complex known as iLipase that is retained within the cartridge. The fat in enteral formula is hydrolyzed as it comes in contact with iLipase in the cartridge. Because fats containing LCPUFAs must be hydrolyzed by pancreatic lipase, RELiZORB is particularly important for the intestinal absorption of dietary LCPUFAs. RELiZORB hydrolyzes triglycerides (TGs) in a wide range of commercially available enteral formulas that are used by individuals with EPI and fat malabsorptive disorder (18).

In a recently published randomized study involving 33 patients with CF and EPI receiving EN, the short-term use of RELiZORB was safe, well-tolerated, and significantly increased plasma omega-3 FA levels, systemic markers of fat absorption (5). Because this study involved only a single treatment with RELiZORB, a study to evaluate its longer-term use was needed.

The Absorption and Safety with Sustained use of RELiZORB Evaluation (ASSURE) Study in Patients with Cystic Fibrosis Receiving Enteral Feeding was designed to evaluate safety, tolerability, and efficacy of sustained use of RELiZORB over a 90-day period in patients with CF and EPI using EN as part of their regular nutrition regimen. The omega-3 index used in this study provides a measure of tissue DHA and EPA levels and is a reliable estimation of LCPUFA absorption in patients with CF (15,19). This index was first used as a predictor of cardiovascular outcomes and has been applied to studies of patients with CF (19). As a measure of nutritional health, the omega-3 index has the advantage of being a measure of long-term tissue LCPUFA concentrations, being unchanged by patient fasting or fed state, and being easily measured through blood collection (15,20).

## METHODS

The ASSURE trial was a prospective, single-arm, multicenter, open-label study of RELiZORB cartridge use with EN tube feedings in patients with CF over a 90-day study period (ClinicalTrials.gov Identifier: NCT02750501). The study was conducted between July 20, 2016, and March 30, 2017. Patients were eligible for the study if they were ≥4 years of age, had a confirmed diagnosis of CF, a documented history of EPI, were taking enteral formula a minimum of 4 times per week, were using PERT, and were consuming an unrestricted fat diet. Patients were excluded if they had uncontrolled diabetes mellitus, signs and symptoms of hepatic cirrhosis, portal hypertension, or significant liver disease (defined as liver transaminases greater than 3x the upper limit of normal or total bilirubin greater than 1.5x the upper limit of normal), received a lung or liver transplant, active cancer for which they were currently receiving treatment, Crohn disease, celiac disease, diarrheal illness unrelated to EPI (eg, infectious gastroenteritis, sprue, lactose intolerance, or inflammatory bowel disease), history of fibrosing colonopathy or recurrent distal intestinal obstructive syndrome. The study protocol and informed consent forms received institutional review board approval.

The 90-day study treatment period was preceded by a 7-day observation period and a 7-day run-in period (Supplemental Digital Content 1). Throughout the observation period (day −14 to day −8), patients received their usual EN tube-feeding regimens, including PERT. The patients then entered a study run-in period (day −7 to day −1), during which they each received their usual volume of tube-fed enteral formula (minimum 500 mL and maximum 1000 mL per feeding) for at least 5 days before the treatment period. All patients used the same enteral formula, Peptamen 1.5^;^ (Nestlé Nutrition Inc, Florham Park, NJ), in place of their usual formula. Peptamen 1.5, which contains 14 g of fat per 250 mL (70% medium-chain TGs [MCTs] and 30% long-chain TGs; LCTs but no DHA or EPA added) (21), was chosen because it is a commonly used semi-elemental formula in patients with CF and is similar to formula with a similar fat content used during the 90-day study period.

Throughout the study treatment period with RELiZORB, at least 5 days a week, participants received 500 to 1000 mL of Impact Peptide 1.5, (Nestlé Nutrition). Impact Peptide 1.5 contains 15.9 g of fat per 250 mL (50% MCTs and 50% LCTs), as well as 15.9 g/L of DHA and EPA (22). It is an enteral formula like Peptamen 1.5 but with a higher ratio of long-chain to medium-chain TG (21,22). A single RELiZORB cartridge was used for each overnight feed. During all phases of the study, participants continued their pre-study regimens of PERT use with oral meals and snacks. During the observation and run-in periods, participants used PERT with EN feedings, but during the 90-day treatment period, participants were not allowed to use PERT with overnight EN feedings.

All efficacy outcomes were measured at observation initiation (day –14), before RELiZORB initiation (day 0), and at 30-day intervals during the RELiZORB treatment period study (days 30, 60, and 90). The primary efficacy outcome measure was the change in omega-3 index, which is a measure of the percentage of total DHA plus EPA (DHA+EPA) relative to the total fatty acid (FA) composition present in erythrocyte membranes. Secondary efficacy outcomes included changes in plasma and erythrocyte membrane composition (%) of total EPA, total DHA, and omega-6 to omega-3 FAs (a key marker of inflammation) as well as plasma concentrations of total DHA+EPA. Plasma concentrations (μg/mL) of total DHA and total EPA were measured using a validated ultra-high-performance liquid chromatography (UHPLC) method by PPD Laboratories (Richmond, VA) adapted from protocols described Bowen et al (23). Total EPA+DHA was calculated as the sum of the concentrations of total DHA and total EPA. FA composition (%) in both erythrocytes and plasma was measured using gas chromatography-mass spectrometry (GCMS) methods by OmegaQuant (Sioux Falls, SD) as described by Potala et al (24). Exploratory efficacy outcomes included changes in plasma levels of fat-soluble vitamins A, D, and E, serum total protein, pre-albumin, albumin, and transferrin. Changes in weight gain and standardized body weight and body mass index (BMI) were also examined. Weight and BMI were normalized to age- and sex-specific *z* scores of healthy individuals aged 2 to 20 years using data from the Centers for Disease Control and Prevention.

Safety and tolerability outcomes included the frequency and severity of adverse events (AEs) and unanticipated adverse device effects (UADEs), incidence of GI symptoms, clinical and laboratory findings, vital signs, and use of concomitant medications. AEs and UADEs were classified as related to RELiZORB if there was strong medical evidence of an association between the AE or UADE and RELiZORB use. Baseline measurements were defined for safety assessments as the last non-missing measurement taken before the administration of RELiZORB, for GI symptoms as symptoms recorded in the GI-symptom diary during the run-in period, and for efficacy assessments as those taken on day 0, or on day –14 if missing result for day 0.

During the treatment phase of the study, from day 0 to 90, any UADEs were identified during scheduled study visits, through spontaneously reported medical complaints, and through investigations of non-scheduled healthcare visits. All AEs were coded using the Medical Dictionary for Regulatory Activities (MedDRA Version 18.0) and summarized based on the treatment at the time the event started. Participants recorded GI symptoms and concomitant medications in a study-specific symptom diary for 7 days before the day 0 visit and again before visits for days 30, 60, and 90 (Supplemental Digital Content 1).

Statistical analysis was conducted using SAS software version 9.3 (SAS, Inc, Cary, NC). Sample size calculations estimated that 30 participants would be needed for 80% power to detect a minimum effect size of 0.53 based on a maximum DHA standard deviation (SD) of 1.5%. Assuming the same effect for DHA+EPA as for DHA alone with 30 participants, the study would have 80% power to detect a 0.8% absolute increase or 30% relative increase in DHA+EPA levels from baseline to study day 90. Study participants were included in the statistical analysis if they used RELiZORB at least once during the study. All efficacy endpoints were analyzed using mixed-model repeated-measures models, testing for least-square (LS) mean differences between baseline and post-baseline visits, where study visit is a categorical effect and study participant is a random effect.

## RESULTS

Of 49 patients screened, 44 enrolled as participants in the study, 5 patients discontinued before the RELiZORB treatment period (day 0), 39 used RELiZORB at least once, and 36 patients completed the study (Supplemental Digital Content 2). The 39 participants ranged in age from 5 to 33 years, had a mean duration of enteral tube feeding of 6.2 years (range 0.7–17.5 years), a mean BMI of 17.7 kg/m^2^, and 9 (23.1%) had cystic fibrosis-related diabetes (Table 1). Each feeding delivered a mean (SD) volume of 756 (186) mL at a rate of 120 (77) mL/h, and 5.8 PERT (3.1) capsules were used per EN feeding. Over the course of the 90-day experimental treatment period, participants used RELiZORB for a mean (SD) of 68.4 (18.43) days with a mean (SD) days per week of enteral tube feedings of 5.3 (1.2) days and a mean (SD) formula volume per day of 755.9 (145.16) mL.

**FIGURE 1:**
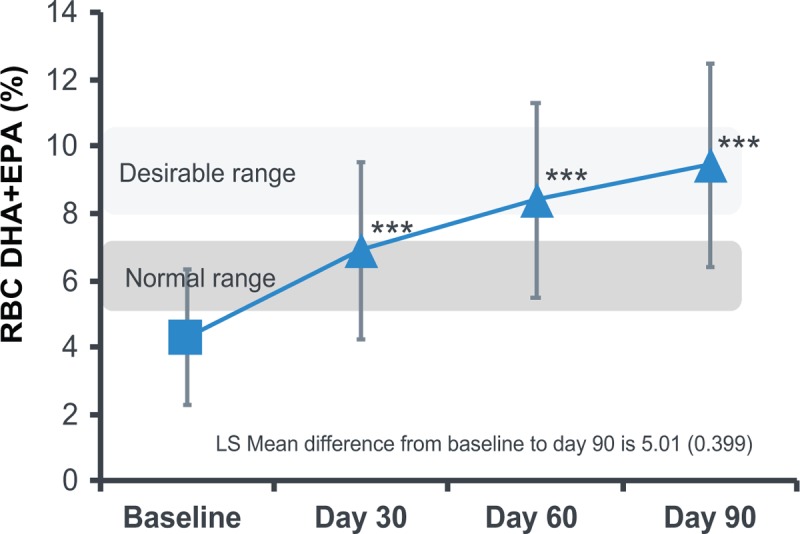
Changes in erythrocyte membrance fatty acid composition (%) for omega-3 index (ITT population)—composition of FA in erythrocyte membranes was measured by gas chromatography-mass spectrometry (GC/MS) (OmegaQuant, LLC, Sioux Falls, SD).

The omega-3 index increased from a baseline value of 4.4%, which was below the target range of 8%, to 8.4% at 60 days and 9.4% at 90 days (*P* < 0.001 for each increase from baseline to 60 and 90 days). (Fig. 1). The magnitude and significance of omega-3 index increases were similar in the 2 younger age groups (≤12 years, 13–18 years) to the overall findings, but in adults (≥19 years) the differences were not statistically significant at day 60 (*P *= 0.051), likely because of the small sample size (n = 5) (Supplemental Digital Content 3).

All secondary efficacy outcomes changed significantly from baseline to each post-baseline visit. Values increased for the composition (%) of erythrocyte membrane total DHA and total EPA, and plasma concentrations of total DHA, total EPA, and total DHA+EPA. The omega-6 to omega-3 FA ratios decreased in both erythrocyte membranes and plasma (Table 2). All exploratory efficacy outcomes including serum levels of fat-soluble vitamins A, D, and E in plasma total protein, pre-albumin, albumin, and transferrin were within normal ranges at study entry and remained so throughout the 90-day study treatment period. The notable exception among the exploratory outcomes examined was weight and age- and sex-specific *z* score means of BMI which were below target goal at study entry and did not change significantly over the course of the study period (data not shown).

In the RELiZORB treatment period, at least 1 AE was reported by 29 (74%) patients with greatest incidence for respiratory (46%), infections (20%), and investigations (20%), which included decreased forced expiratory volume, pulmonary function test, and vitamin D levels. Only 1 AE reported as constipation was judged by the principal investigator (PI) to be possibly device related. No AEs resulted in discontinuation of enteral feeding. At least 1 UADE was reported by 10 of 39 (25.6%) participants, including 2 participants with infections and 8 with respiratory, thoracic, and mediastina disorders, but none were classified as being related to the RELiZORB use. Only 1 of these participants discontinued enteral feeds because of their UADE, which was an infective pulmonary exacerbation of CF requiring hospitalization unrelated to RELiZORB use. Using the 7-day GI symptom diary, a decreasing number of participants reported symptoms over the course of the study. Abdominal pain and gas were the most commonly reported symptoms (Table 3). There were no deaths in the study.

## DISCUSSION

Findings from the ASSURE study demonstrate that 90 days of RELiZORB use with overnight EN feedings is safe, well-tolerated, and associated with improvements of LCPUFA levels in patients with CF and EPI. Despite having used overnight EN feedings for an average of 6 years, patients entering the study had low plasma and erythrocyte levels of DHA and EPA reflecting a poor nutritional status. During the study, participant DHA and EPA levels normalized in both the plasma and erythrocyte membranes indicating improvement in fat malabsorption with the use of RELiZORB. Serum protein and fat-soluble vitamin levels were normal at study baseline and did not change significantly with 90 days of RELiZORB suggesting that low FA levels as well as low BMI were the main nutritional deficiencies observed in the study population.

As in the currently reported ASSURE study, participants in the previous RELiZORB study were deficient in DHA and EPA at study entry even though they had been taking supplemental EN feedings for over 6 years (5). In both studies, Impact Peptide 1.5 was used as the formula with RELiZORB because it has a relatively high percentage of fat as long-chain TGs (MCT:LCT 50:50), which are more difficult than medium-chain TGs to absorb in the absence of pancreatic lipase, making it a more rigorous test of RELiZORB than other enteral formulas (5,22). In the previous short-term study, plasma DHA and EPA levels increased significantly with levels 2.8 times higher than controls in the randomized cross-over phase (5). In the ASSURE study, omega-3 FA erythrocyte levels increased with longer-term RELiZORB use, supporting the role of RELiZORB in normalizing deficient DHA and EPA levels and maintaining them over longer periods when RELiZORB was utilized with overnight EN feeds.

The findings of this longer-duration study also confirm the favorable safety and efficacy findings of the previous clinical RELiZORB studies (5). In a previous study, GI symptoms were reported less frequently and with less severity in participants using RELiZORB. Participant-reported appetite was also better when using RELiZORB than just oral PERT (5). In the ASSURE study, the frequency of RELiZORB-related UADEs was low, despite a high overall frequency of AEs, which were primarily respiratory events unrelated to the study treatment. In addition, the number of participants reporting GI symptoms decreased in the group using RELiZORB throughout the ASSURE study.

It is not possible to draw definitive conclusions from the current and previous RELiZORB studies about the influence of RELiZORB use on changes in patient anthropometric measurements. While weight, BMI z-scores and percentiles were not significantly different from baseline to 90 days, 61% (20/33) patients in the ASSURE study had improvements in weight *z* scores and percentiles over the course of the study. Furthermore, recent data based on retrospective case series of patients from CF treatment centers has reported that RELiZORB use was associated with improvements in weight gain, weight for age *z* scores, the ability to meet or exceed age appropriate weight goals while simultaneously reducing GI symptoms associated with fat malabsorption (25–27).

While this study has several strengths, it also has some limitations. It is a relatively small study, but represents 1% of the estimated 3600 US patients with CF who receive supplemental enteral tube feedings (4). A strength of the study is its long duration compared to the previous 7-day study of RELiZORB use (5). In addition, the study was strictly open label and was not intended to compare outcomes between participants who did and did not use RELiZORB. Furthermore, other than EN, dietary intake was not recorded during the study, limiting the ability to assess whether oral caloric intake was adequate to lead to weight gain. Additionally, the study may not have been long enough to observe an increase in body weight or BMI for patients in a relatively fat-starved condition (13). Because the current study did not measure body tissue composition, it is unknown whether study participants improved their tissue composition without changing body weight or size. It may turn out that nutritional health may be more accurately measured using biomarkers other than the traditional body weight and BMI (28,29). Body tissue composition, including fat mass, fat-free mass, and lean body mass may be more important to overall health than body size or mass (23). It is likely that body tissue composition is an indirect measure of important cellular and molecular processes that may be directly measured using molecules such as LCPUFAs, which are essential building blocks of cell membranes and play important roles in cell and tissue function throughout the body (30–32).

Given the strengths of the ASSURE study and the finding of improved LCPUFA levels with RELiZORB use in patients with CF and EPI on EN tube feedings, RELiZORB is a safe and effective option for increasing fat absorption in this population. Patients with CF and EPI may have poor nutritional status despite long-term use of supplemental EN feedings. This study establishes the safety and tolerability of RELiZORB and shows its potential to normalize fat absorption, improve symptoms commonly associated with fat malabsorption and enhance nutritional status in patients with CF receiving EN feedings.

## Supplementary Material

Supplemental Digital Content

## Supplementary Material

Supplemental Digital Content

## Supplementary Material

Supplemental Digital Content

## Figures and Tables

**TABLE 1:** Absorption and Safety With Sustained Use of RELiZORB Evaluation study demographics and baseline characteristics (ITT population)

Parameter	ITT population (n = 39)
Age, y	
Mean (SD)	13.8 (5.4)
Min, Max	5, 33
Age category, y	
≤12	17 (43.6%)
13–18	17 (43.6%)
≥19	5 (12.8%)
Gender	
Male/female	24 (61.5%)/15 (38.5%)
Weight, kg	
Mean (SD)	40.83 (12.3)
Height, cm	
Mean (SD)	149.84 (18.9)
BMI, kg/m^2^	
Mean (SD)	17.68 (1.8)
CFRD category	
CFRD	9 (23.1%)
Non-CFRD	30 (76.9%)

BMI = body mass index; CFRD = cystic fibrosis-related diabetes; ITT = intent-to-treat.

**TABLE 2:** Changes in docosahexaenoic acid, eicosapentaenoic acid, docosahexaenoic acid+eicosapentaenoic acid. and ω-6/ω-3 ratio in plasma

	Baseline, n = 36	Day 30, n = 36	Day 60, n = 36	Day 90, n = 36
DHA, μg/mL	50.33 (34.1)	102.64 (39.3)	96.77 (36.4)	102.36 (36.3)
EPA, μg/mL	22.41 (21.1)	65.34 (45.9)	64.01 (49.6)	64.09 (41.5)
Total DHA+EPA, μg/mL	72.73 (52.1)	167.99 (82.5)	160.78 (83.2)	166.46 (73.7)
ω-6/ω-3 ratio	11.52 (4.3)	5.31 (2.5)	5.63 (3.3)	5.23 (3.1)

Plasma concentrations of total DHA and EPA were measured by ultra-high-performance liquid chromatography (UHPLC) (PPD LLC, Richmond, VA). Total DHA+EPA were calculated by adding the concentrations of total DHA and EPA. DHA = docosahexaenoic acid; EPA = eicosapentaenoic acid.

**TABLE 3:** Gastrointestinal adverse events for ITT population (n = 39)

Number of subjects with incidence of GI symptoms	PERT + Usual enteral formula	PERT + Peptamen 1.5	90-day open-label treatment period RELiZORB with Impact Peptide 1.5		
	Observation period	Run-in period	Day 30	Day 60	Day 90
Any symptom	23 (59.0%)	22 (56.4%)	16 (41.0%)	17 (43.6%)	12 (30.8%)
Abdominal pain	12 (30.8%)	12 (30.8%)	11 (28.2%)	9 (23.1%)	7 (17.9%)
Bloating	4 (10.3%)	2 (5.1%)	3 (7.7%)	2 (5.1%)	3 (7.7%)
Constipation	2 (5.1%)	0	0	2 (5.1%)	2 (5.1%)
Diarrhea	4 (10.3%)	6 (15.4%)	3 (7.7%)	4 (10.3%)	0
Gas	16 (41.0%)	16 (41.0%)	9 (23.1%)	11 (28.2%)	8 (20.5%)
Indigestion/heartburn	3 (7.7%)	2 (5.1%)	2 (5.1%)	4 (10.3%)	3 (7.7%)
Nausea	6 (15.4%)	5 (12.8%)	2 (5.1%)	4 (10.3%)	3 (7.7%)
Steatorrhea (fatty stool)	2 (5.1%)	3 (7.7%)	2 (5.1%)	2 (5.1%)	0
Vomiting	4 (10.3%)	4 (10.3%)	2 (5.1%)	2 (5.1%)	0

GI = gastrointestinal; ITT = intent-to-treat; PERT = pancreatic enzyme replacement therapy.
