# Supplementary figures and images for: Absorption and Safety With Sustained Use of RELiZORB Evaluation (ASSURE) Study in Patients With Cystic Fibrosis Receiving Enteral Feeding
Source: J Pediatr Gastroenterol Nutr. 2018 Aug 1;67(4):527–32. doi: 10.1097/MPG.0000000000002110 (PMC6155360; doi:10.1097/MPG.0000000000002110)

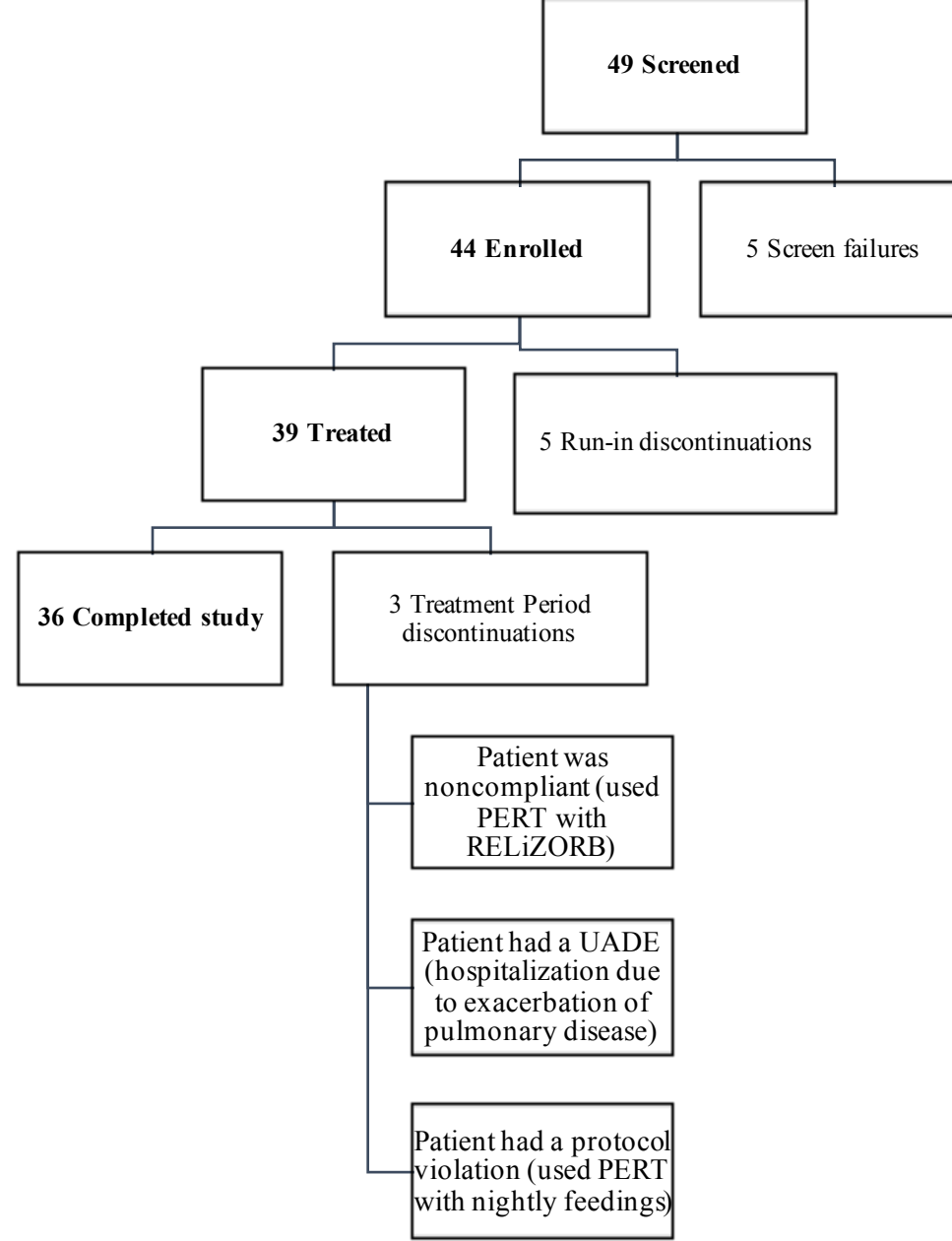

**Supplemental Digital Content 2 : ASSURE CONSORT Flow Diagram**

Supplement: Supplemental Digital Content [file jpga-67-527-s002.pdf]
